# Price and affordability of direct-acting antiviral regimens for hepatitis C virus in the United States

**DOI:** 10.1186/s13027-016-0071-z

**Published:** 2016-05-16

**Authors:** Elana S. Rosenthal, Camilla S. Graham

**Affiliations:** Clinical Research Directorate/Clinical Monitoring Research Program, Leidos Biomedical Research, Frederick National Laboratory for Cancer Research, Frederick, MD USA; Division of Infectious Diseases, Beth Israel Deaconess Medical Center and Harvard Medical School, Boston, MA USA; Trek Therapeutics, PBC, Cambridge, MA USA

**Keywords:** Hepatitis C virus, Cost-effectiveness, Pharmaceutical pricing, Direct-acting antivirals

## Abstract

Hepatitis C virus is a serious infection causing cirrhosis, liver cancer, and death. The recent development of direct-acting antivirals has dramatically improved tolerability of treatment and rates of cure. However, the high price of these medications has often limited access to care and resulted in rationing of medications in the United States to those with advanced liver disease, access to specialist care, and without active substance use. This review assesses the way pharmaceutical prices are established and how pricing of directly acting antiviral regimens in the United States has impacted access to treatment for hepatitis C virus.

## Background

Hepatitis C virus (HCV) is a serious infection that chronically infects approximately 135 million people worldwide [1]. There is ongoing acute transmission of HCV, especially in young people who inject drugs [2], and human immunodeficiency virus (HIV) infected men who have sex with men [3].

Chronic infection with HCV can lead to cirrhosis, liver cancer, and death, and is the leading cause of liver transplantation in the United States [4]. HCV is treatable, and the goal of treatment is to achieve a sustained virologic response (SVR), considered to be a functional cure (absence of plasma HCV RNA 12 weeks after completing therapy). Historically, treatment of HCV using interferon-alfa involved significant toxicities and poor rates of SVR, particularly in patients with HIV and/or black race.

The advent of direct-acting antivirals (DAA) has been revolutionary in the advancement of HCV treatment. DAAs have few side effects, short durations of treatment, and high SVR (see Table 1). In addition, they are effective regardless of race, gender, or HIV status, leaving few barriers to treatment [5, 6]. Therefore, in HCV infected individuals, DAAs have the potential to lower mortality, improve quality of life, reduce long-term costs of complications and interrupt the current global HCV epidemic [7].

**Table 1:** Comparison of Regimens for Treatment of HCV genotype 1

Regimen	Duration	Side effects	Contraindications	Characteristics that decrease SVR
Peg-IFN + Ribavirin	48 weeks	Fatigue 65 % Headache 43 % Pyrexia 41 % Myalgia 40 % Anxiety 33 % Alopecia 28 % Neutropenia 27 % Rigors 25 % Depression 20 %	Child-Pugh B or C Autoimmune hepatitis Pregnancy or a pregnant partner Neuropsychiatric illness	HIV co-infection Black IL28B-nonCC Cirrhosis
Peg-IFN + Ribavirin + Telaprevir	24 weeks	As above plus Rash 56 % Pruritis 47 % Nausea 39 % Anemia 36 %	Child-Pugh B or C Autoimmune hepatitis Pregnancy or a pregnant partner Neuropsychiatric illness	HIV co-infection Black IL28B-nonCC Cirrhosis
Ledipasvir/Sofosbuvir (Harvoni)	12 weeks or 24 weeks	Headache 14 % Fatigue 13 % Nausea 7 % Insomnia 5 % Diarrhea 3 %	Severe renal impairment	None
Ombitasvir/Paritaprevir/ritonavir/Dasabuvir (Viekira Pak) Ribavirin	12 weeks	Fatigue 34 % Nausea 22 % Pruritis 18 % Skin reactions 16 % Insomnia 14 % Asthenia 14 %	Severe hepatic impairment Pregnancy or a pregnant partner	None
Grazoprevir/Elbasvir (Zepatier) +/- Ribavirin	12 weeks or 16 weeks	Fatigue 5 % Abdominal pain 2 % Diarrhea 2 % Depression 1 % Irritability 1 %	Child-Pugh B or C In patients on Ribavirin: Pregnancy or a pregnant partner	Baseline NS5A polymorphisms

Above data from package inserts for products

A major obstacle to wide use of DAAs remains the high price of these drugs, preventing access to HCV treatment for those in need. Despite scientific information and guideline recommendations to the contrary, prices set by pharmaceutical companies have resulted in payer driven rationing of care in the United States [8]. This has largely limited access to treatment to a subset of patients with advanced liver disease and without ongoing substance use. This approach will not reduce ongoing transmission of HCV or prevent risk of advanced liver disease and liver cancer [9].

In the treatment of HCV, as in many diseases, pricing of treatment has a significant impact on patient care. Therefore, it is important to understand the price we pay for medical care. In this review, we will assess the way pharmaceutical prices are established, as well as the true cost of DAA treatment of HCV in the United States.

## Drug pricing

**Fig. 1:**
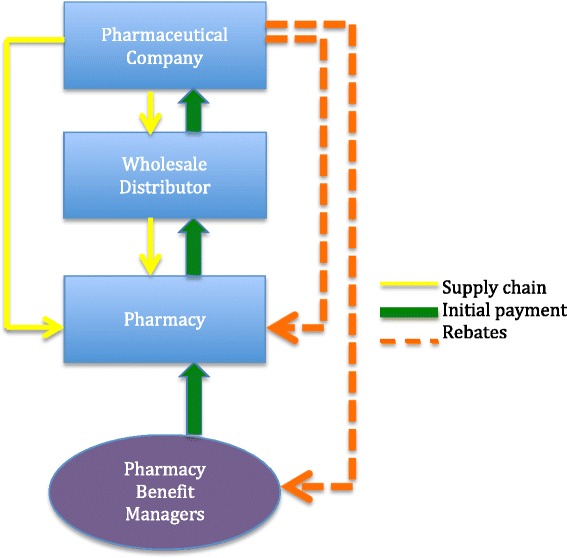
Supply chain, initial payment, and rebates for pharmaceutical drugs. In the pharmaceutical supply chain (*yellow line*), drugs are moved from the pharmaceutical company to the wholesale distributor to the pharmacy or directly from the pharmaceutical company to the pharmacy. In the payment pathway, initial payment (*green line*) is essentially the reverse of the supply chain, with the addition of the pharmacy benefit managers acting as a third party aiding in financial negotiations. At each stage a percentage of payment is retained. Discounts on the initial payment can be made based on purchasing power and prompt pay. Rebates (*orange line*) are money that is paid back by the pharmaceutical company after initial payment. Pharmaceutical companies give rebates for preferential use the manufacturer’s drug, high volume of drug sales, and based on established government regulations

The pricing of drugs is impacted by many factors, including market competition, presence of generics, existing prices of effective treatment and business negotiations. Though there is little transparency in the process, there are key concepts that are important to understand (see Fig. 1). Of note, for the purposes of this review we will only discuss brand name drugs and focus on the U.S. landscape.

### Supply chain

The supply chain demonstrates the transfer of possession of the drug. In the supply chain, there are three main entities, the pharmaceutical company, wholesale distributors, and pharmacies.

**Pharmaceutical companies** are generally large, for-profit, multinational corporations that do expensive research and drug development. Most drugs are sold by pharmaceutical companies to wholesale distributors. **Wholesale distributors** store products, manage the inventory, and subsequently distribute the supply to pharmacies and other medical facilities. They largely act as a supply chain middleman between pharmaceutical companies and pharmacies.

**Pharmacies** are responsible for safe storage of drug products, dispensing medications to patients, and managing billing and payment between patients and insurance companies. Non-retail providers such as hospitals, clinics, and federal facilities purchase the majority of their products from wholesale distributors. In contrast, chain and food store pharmacies often purchase directly from the manufacturer as they have in house capabilities for warehousing and managing inventory. Pharmacies operate the last step in the supply chain, delivering the medication to the patient [10–12].

### Payment pathway

Though the supply chain is fairly linear, the payment pathway is less straightforward. In terms of financial transactions, the members of the supply chain continue to be involved, with the addition of **Pharmacy Benefit Managers (PBM).** PBMs are a third party that helps negotiate financial transactions on behalf of their clients, generally insurance companies. Payments are made in two ways: 1) the initial payment and 2) rebates from the pharmaceutical company after payment has been made.

The initial payment pathway is essentially the reverse of the supply chain. The PBM, on behalf of the insurance company, pays the pharmacy. The pharmacy in turn pays the wholesale distributor, and the wholesale distributor pays the pharmaceutical company. At each stage a percentage of payment is retained. **Discounts** on the initial payment are given based on large purchasing power and prompt payment [10–12].

**Rebates** are money that is paid back by the pharmaceutical company after initial payment. Therefore, the final price paid to the pharmaceutical company is the initial payment it receives, minus the subsequent rebates. Pharmaceutical companies give rebates to PBMs, pharmacies, and government organizations. Formulary payments are rebates made to PBMs for giving the manufacturer’s drug some preference over similar drugs by inclusion in the PBM’s formulary, often with a lower copay or fewer restrictions than competitor drugs. Market share rebates are given after a PBM demonstrates that they were able to successfully direct consumers to the manufacturer’s product over the competitor’s product. Discounts are also given after a predetermined volume of drug sales have been achieved. Prompt pay rebates are given when pharmaceutical companies receive their initial payment within a defined time period [10–12].

The amount of money paid during initial payment and rebate is generally not publically known due to the lack of transparency in the pharmaceutical company’s negotiations with payers. However, the basis for negotiation starts with the publically available list price set by the pharmaceutical company, called the **Wholesale Acquisition Cost (WAC)**. In setting the WAC, a predominant consideration is what the market will bear. Drugs are priced based on what pharmaceutical companies believe can maximize profits, rather than what price will cover prior investment and increase access to consumers. Consideration is also given to cost-effectiveness models, budget impact models, and benchmarking against similar regimens. Further they assess expectations of shareholders, cost of research and development, manufacturing and marketing [10–12].

### United States special pricing considerations

In the United States, certain government groups get special consideration for initial pricing and rebates as a condition of including drugs in their formularies. The basis for this pricing often takes into account the **Average Manufacturer Price** (AMP), the average price paid by wholesale distributors to pharmaceutical companies. This amount is not publically available.

**Medicaid** is a government subsidized healthcare program available for individuals with low-incomes. Though subsidized by the federal government, Medicaid is run independently in each state. The availability of Medicaid coverage was recently expanded in 31 states under the **Affordable Care Act** to allow greater access to healthcare. For new brand name drugs, Medicaid receives a minimum drug rebate of 23.1 % of the AMP, or the difference between the AMP and the lowest price paid by a private sector payer (known as “best price”), whichever amount is greater. As a condition of having drugs covered by Medicaid, **340B pharmacies**, non-federal entities servicing indigent populations, receive drugs priced equivalent to Medicaid pricing.

**Medicare** is a federally subsidized insurance available to Americans aged 65 or older and younger people with disabilities. **Medicare Part D** is the prescription drug benefit for Medicare beneficiaries, established in 2003 as part of the Medicare Modernization Act. In order to garner support from the pharmaceutical industry to pass this legislature, a provision was included prohibiting the government from negotiating with pharmaceutical companies over Medicare drug prices. An estimated $15–16 billion could be saved annually if negotiations were permitted [13]. Part D plans are managed by private insurance companies that set formularies and tiered pricing of drugs. Private plans negotiate discounts, but these are often less than those negotiated by federal agencies [14].

The **Federal Supply Schedule (FSS)** sets drug prices for the Veterans Health Administration, Department of Defense, Public Health Service, Indian Health Service, and federal prisons. The initial price paid by the FSS has to be under the federal ceiling, which is the AMP minus 24 %. With further negotiations for rebates, the FSS often pays even less [10–12].

Many groups lack special consideration for discounts and rebates. In particular, state prisons and jails do not fall under the auspices of the FSS and do not receive Medicaid related rebates. State jails and prisons also contribute to calculations of best-price, this further limits the discounts pharmaceutical companies are willing to provide. Given they lack the negotiating leverage of larger organizations, these entities pay among the highest prices for pharmaceuticals [10–12].

## Current DAA pricing

Though the concept of rebates is public knowledge, the amount of money pharmaceutical companies receive for drug sales is considered to be a confidential business contract. As stated previously, the WAC is publically available, however generally does not reflect the actual price paid for drugs.

### United States pricing

In the United States, sofosbuvir (Sovaldi) was approved in 2013 and the WAC was set at $84,000 for a 12-week course of treatment. Subsequent pricing of DAAs was similarly high (see Table 2). Though the $1,000 per pill price tag established by sofosbuvir set a high bar for pricing of DAAs, the emergence of market competition has allowed for greater discounts and rebates. When Viekira Pak was approved, AbbVie contracted with Express Scripts, one of the largest PBMs in the U.S. In exchange for removing Harvoni from their formulary, Express Scripts was able to receive a course of Viekira Pak for approximately $51,000–$66,000 [15]. Currently, 80 % of the market is exclusive to one of these two drugs, and the average negotiated discount is 46 % off of the WAC [16].

**Table 2:** Wholesale acquisition cost of direct-acting antivirals

Direct-acting antiviral	Pharmaceutical company	WAC for 12 week course
Sofosbuvir (Sovaldi)	Gilead sciences	$84,000
Ledipasvir/Sofosbuvir (Harvoni)	Gilead sciences	$94,500
Ombitasvir/paritaprevir/ritonavir + Dasabuvir (Viekira Pak)	AbbVie	$83,319
Daclatasvir (Daklinza) + Sofosbuvir (Sovaldi)	Bristol-Myers Squibb and Gilead	$147,000
Grazoprevir/Elbasvir (Zepatier)	Merck	$54,600

### Non-United States pricing

The United States pays a disproportionate amount for pharmaceuticals, even in comparison to other developed nations. This in part reflects the fact that countries with single payer healthcare systems are better able to negotiate affordable rates with pharmaceutical companies, and many have better mechanisms to control costs. In the case of sofosbuvir (SOF), the U.S. price for 12-weeks is $84,000, whereas the UK price is $54,000, and the price in Spain is $25,000.

In low and middle-income countries, where over 80 % of HCV infected individuals reside, there is a degree of cost sharing and subsidizing by developed countries because the market will not bear higher price points. Pharmaceutical companies have several strategies to increase access in these countries without losing control of the product. Tiered pricing categorizes countries by per capita gross national income, and selects prices based on tier. For instance, in Egypt, SOF has been priced at $900 for a 12 week course of treatment. However, even at lower prices, poor and uninsured individuals in these countries are often unable to afford these medications [17].

Voluntary licensing enables the patent-holder to license one or more manufacturing company to produce generic medications, usually in exchange for a royalty [18]. In these cases, the extent to which prices can be reduced largely relies on the terms agreed upon between the patent holder and manufacturer. For example, terms may specify allowed price ranges, or set limits on the number of patients or categories of patients eligible to receive these generic treatments [19]. Gilead Sciences currently holds agreements with 11 Indian companies to manufacture generic SOF, ledipasvir/SOF, and velpatasvir/SOF for 101 developing countries [20]. For distribution of daclatasvir, BMS currently employs a tiered pricing scheme. However, notably, BMS has entered into the first ever licensing agreement for a HCV Medicines Patent Pool, which will provide daclatasvir licenses, free of royalties, for 112 developing countries [21]. In China and India, patent applications for SOF were denied altogether. This will allow generic manufacturers full freedom to produce and distribute medications, as well as increase the potential for market competition to drive down prices to an affordable level [22, 23]. Finding ways to allow patients in low and middle income countries to access DAAs remains one of the major challenges to eradicating HCV.

## Cost effectiveness

A major consideration of medical therapy is not only the cost of treatment, but whether a given treatment is cost effective. While current DAA prices are exorbitant, the cost of cure is comparable to that of interferon based therapy (see Table 3). Undoubtedly this equivalent cost of cure was taken into account when setting the price for these medications.

**Table 3:** “Standard of care” regimens for non-cirrhotic, treatment naïve patients with HCV Genotype 1, and cost per SVR

Regimen	SVR rate	WAC price	Cost per SVR
Pegasys + Ribavirin x48 weeks	41 %	$41,758	$101,849
Telaprevir + PegIFN + Ribavirin x24 weeks	75 %	$86,843	$115,791
Sofosbuvir + PegIFN + Ribavirin x12 weeks	90 %	$94,421	$104,912
Sofosbuvir + Ledipasvir x12 weeks	99 %	$94,500	$95,454
Grazoprevir + Elbasvir x12 weeks	94 %	$54,600	$58,085

Above data from package inserts for products

Cost effectiveness models help make an objective calculation of the price at which a given medication is worth the beneficial effect it has on health. Numerous cost effectiveness studies have been done in HCV, and in general, treatment with DAAs is cost-effective relative to previous stand-of-care for most US populations [10, 24]. A few studies are highlighted. Najafzadeh et al. developed a discrete-event simulation to simulate natural history and progression of liver disease among treatment naïve individuals with chronic HCV infections due to HCV genotype 1, 2, or 3. This model utilized a hypothetical cohort of 10,000 patients with baseline characteristics emulating the US population, and disease progression based on the literature. Five treatment strategies were considered: 1) PEG/RBV + boceprevir, 2) PEG/RBV + SOF, 3) SOF + simeprevir, 4) SOF + daclatasvir, or 5) SOF/LDV. Their model found that treatment of patients with genotype 1 was very cost-effective, at $12,825 per quality-adjusted life year (QALY) gained compared to peginterferon(peg-IFN)/ribavirin/boceprevir, even when estimating the cost of treatment without discounts or rebates. Further, a 12-week regimen of ledipasvir/SOF priced at $76,500 could be cost saving for patients with genotype 1. As expected, they found that cost-effectiveness was greatest in those patients with increased fibrosis and younger age [25].

Leidner et al. examined cost effectiveness of DAA therapies, stratifying by stage of fibrosis at time of treatment [26]. They modeled a closed population of adults, with all individuals chronically infected prior to analysis, no entry into population over time, and possible exit due to death. The model accounted for the potential for fibrosis stage to progress from year to year, and the time horizon modeled was the lifetime of the population. Future outcomes, costs and QALYs, were discounted 3 % annually. This model assumed treatment naïve status at entry, but allowed for retreatment in those who failed initial treatment. In their analysis they found that for a hypothetical 55 year old patient treated at a cost of $100,000, treatment of the patient at F0, F1, and F2 yielded cost-effectiveness ratios of $242,900, $174,100, and $37,300 respectively. In order to achieve a $100,000/QALY cost-effectiveness ratio for treatment at F0, cost of treatment would have to be $42,400 or less. Therefore they concluded that immediate treatment of HCV-infected individuals with moderate to advanced fibrosis was cost-effective, but delaying treatment for patients with minimal fibrosis may be reasonable until lower priced treatments are available [26]. Similarly, Linas et al. evaluated cost-effectiveness of treatment of individuals with HCV genotype 2 or 3 with SOF/RBV [27]. In their analysis, they found that SOF based regimens were cost-effective in patients with prior treatment experience or cirrhosis, but were not cost-effective with the current cost of treatment for individuals who were treatment naïve without cirrhosis. However, it must be noted that individuals with HCV genotype 2 or 3 had significantly higher rates of SVR when treated with SOF/velpatasvir compared to SOF/RBV [27–30]. Given the imminent approval of this regimen, the cost-effectiveness of treating patients with genotype 2 or 3 infection will need to be reassessed.

Though these models are helpful in understanding the cost-effectiveness of given treatments, they often fail to take into account considerations other than direct medical costs. In the case of HCV, additional elements not considered in these models include extrahepatic complications of HCV, lost work productivity, as well as the impact of stigma due to the persistence of HCV infection. In addition, the previous models failed to account for the potential benefit of cure as prevention, the concept that transmission of HCV can be attenuated by curing people who are at risk of spreading HCV. This includes people who inject drugs, HIV-infected men who have sex with men, and women of childbearing age [2, 3]. Multiple models have shown that using DAAs to treat people who inject drugs would be effective in decreasing the prevalence of HCV, especially when treatment is initiated at early stages of fibrosis [7].

In contrast to previous models, Van Nuys et al. developed a Markov model to simulate progression of a population susceptible to HCV through infection, and several stages of disease, accounting for the impact of various treatment strategies on disease transmission in individuals with HCV genotypes 1, 2, and 3, [9]. The model allows for uninfected individuals to become infected, and for those cured of HCV to become reinfected. In this way they account for real world concerns regarding reinfection of high risk individuals, and the potential for decreased transmission in populations with lower prevalence of HCV due to large scale treatment efforts. Four treatment scenarios were modeled: 1) “baseline” representing pre-DAA treatment with PEG/RBV to individuals with F3-F4, 2) “treat advanced” modeling the same F3-F4 patients, however using DAAs (LDV/SOF for genotype 1, SOF/RBV for genotypes 2 and 3), 3) “treat all diagnosed” treating all infected and diagnosed patients of all stages of fibrosis, modeling 1.3 million patients treated in the first year, and 4) “treat 5 %” in which 5 % or patients infected with any stage of fibrosis are treated per year, with 125,000 individuals treated in year one. They found that while treating people with advanced fibrosis is beneficial, even more social benefit is derived from treating all patients, including those at early stage disease. This scenario would result in $0.8–1.5 trillion in total social value compared to treatment with interferon-based regimens. Of interest, treating 5 % of the population per year, regardless of degree of fibrosis, yielded greater social benefit than prioritizing F3-F4. The authors concluded that limiting access to treatment to those with advanced disease prolongs transmission and limits social value [9]. These models reflect the implications of the current market, however, as pangenotypic agents become available, and costs continue to fall, overall cost-effectiveness of treatment with DAAs will likely increase.

### Affordability

Though cost-effectiveness is an important consideration in selecting medical therapies, the ultimate affordability of a treatment determines whether it can realistically be utilized. In the case of DAAs, while cost of cure is roughly equivalent to that of interferon-based treatments, the number of patients eligible for treatment is dramatically increased. Though immediate broad implementation of DAA treatment would be money saving over time, the upfront cost of therapy with current pricing is largely believed to be prohibitive. Further, given the United States lacks a single payer system, there is less of an incentive for insurance plans to assume the cost of upfront HCV treatment in order to avert the future costs of complications that may occur after a patient moves to another plan.

## Access to DAA treatment

Given the high price of DAAs and the variable cost of treatment, access to DAA therapy in the U.S. has been disparate across states and insurance plans. However there has been consistency in the efforts to create barriers to receiving this treatment. A study by Barua et al. evaluating Medicaid restrictions for sofosbuvir approval identified that three-quarters of states restricted treatment to individuals with advanced fibrosis (F3-4), and only 8 states did not have any restriction based on level of fibrosis [31]. This recommendation is not supported by FDA labeling or HCV treatment guidelines, and precludes patients from receiving treatment prior to developing significant risks for ongoing liver disease. It was also found that 88 % of states had specific eligibility requirements based on substance use. Half of states required a period of abstinence from alcohol or drugs, some for as long as 12 months. This practice is not supported by scientific data, and, in fact, guidelines recommend against pre-treatment drug screening [32]. Further, two-thirds of states grant approval only when the patient is being treated by a specialist in infectious diseases or gastroenterology, or in consultation with such a specialist. [31] This creates a significant bottleneck in care, and may be prohibitive for patients without favorable insurance or access to specialist care. Many other barriers to care exist, including clinically irrelevant laboratory requirements, and contracts limiting patients to one course of treatment per lifetime (regardless of reason for treatment failure). Drug company patient assistance programs had previously served as a safety net for many of these patients, however, they too have developed prohibitive restrictions on dispensing medications [33]. The result is that many patients with mild to moderate fibrosis, active substance use, and/or poor access to specialty care are excluded from HCV treatment.

### Implications for the future

Barriers to DAA treatment have stemmed from the actions of both pharmaceutical companies and payers. While it is in the interest of pharmaceutical companies to treat as many patients as possible, the high prices set for DAAs have made scale-up of treatment unrealistic. Further, the concern over disproportionate allocation of resources to HCV treatment has resulted in payers enacting significant restrictions to treatment that are not based in guidelines or scientific data. These systemic barriers to care will likely require systemic solutions. The HCV guidelines were recently amended to remove prioritization for special populations, advising early treatment for all patients [32]. To date, lawsuits over denial of care have been filed in five states and likely will emerge in many others. These efforts may help clarify the legality of arbitrary rationing of treatment.

Given price negotiations and rebates are largely impacted by competition and drive for market share, the emergence of new DAAs may result in further cost decreases. Merck’s newest drug combination, grazoprevir/elbasvir, was recently approved by the FDA at a WAC of $54,600 for a 12 week course of treatment. With this dramatically lower price, there is hope that further reductions in cost of HCV care may be possible in the near future. However, experience with HIV and the pricing of antiretrovirals suggests that pharmaceutical companies are able to sustain high drug pricing over decades. Calls for an HCV analog to the AIDS Drug Assistance Program may reflect a more realistic option for improving access to DAAs in the United States [8].

## Conclusion

The development of DAAs has resulted in dramatic improvement in the tolerability and efficacy of treatment of HCV, with profound potential to prevent liver disease, cancer, and death in HCV infected individuals. Prohibitive costs of treatment set by pharmaceutical companies and rationing by insurance companies have resulted in limited access to treatment in the United States. In particular, people with minimal fibrosis or active substance use are being excluded from care, and the potential for cure as prevention is limited. More regulations and transparency are needed to ensure that prices set by pharmaceutical companies are not just cost-effective, but affordable as well. Further, the amount paid for medications is significantly reduced by discounts and rebates that are negotiated with pharmaceutical companies. Therefore, cost-effectiveness analysis taking into account a more realistic cost of treatment is necessary to help educate payers and policy makers about the value of increasing access to hepatitis C treatment.

## Abbreviations

AMP: average manufacturer price
DAA: direct-acting antiviral
FSS: federal supply schedule
HCV: hepatitis C virus
HIV: human immunodeficiency virus
PBM: pharmacy benefit manager
peg-IFN: peginterferon
QALY: quality-adjusted life year
SOF: sofosbuvir
SVR: sustained virologic response
WAC: wholesale acquisition cost
